# Screening for hip dysplasia in congenital muscular torticollis: is physical exam enough?

**DOI:** 10.1007/s11832-014-0572-5

**Published:** 2014-02-28

**Authors:** Elizabeth R. A. Joiner, Lindsay M. Andras, David L. Skaggs

**Affiliations:** Children’s Orthopaedic Center, Children’s Hospital Los Angeles, 4650 Sunset Blvd., Mailstop #69, Los Angeles, CA 90027 USA

**Keywords:** Congenital muscular torticollis, Developmental dysplasia of the hip, Screening

## Abstract

**Purpose:**

An association between congenital muscular torticollis (CMT) and developmental dysplasia of the hip (DDH) has been established in the literature; however, whether the screening of patients with CMT for DDH requires hip imaging remains controversial. The purpose of this study is to determine (1) the coexistence rate of DDH requiring treatment in individuals with CMT and (2) if physical exam alone is sufficient screening.

**Methods:**

A single-center retrospective chart review was performed among 97 consecutive patients between 1/1/2003 and 9/1/2012 with CMT who had hip imaging performed.

**Results:**

12 % (12/97) of patients with CMT had DDH, all requiring treatment. 75 % (9/12) of the patients with DDH had an abnormal clinical exam. Of the three patients with DDH and a normal clinical exam, two patients were presenting for a second opinion after being treated for DDH prior to evaluation. 90 % (9/10) of patients with DDH at the time of presentation had an abnormal hip exam. All 12 patients with hip dysplasia were referred for DDH or DDH with CMT. There were no patients who were referred for CMT alone that had DDH.

**Conclusions:**

In the care of a patient with CMT, it is important that the clinician remains vigilant about screening for DDH. An ultrasound or radiograph of the hips should be strongly considered as part of the evaluation of a child with CMT.

Level of evidence: IV.

## Introduction

A relationship between congenital muscular torticollis (CMT) and developmental dysplasia of the hip (DDH) was first described by Coventry and Harris [1], followed soon after by the report of a 14.8 % coexistence rate of DDH in patients with CMT by Iwahara and Ikeda [2]. There have since been many reports in the literature that confirm this relationship, although the coexistence rate widely varies from 0 to 20 % [1–19]. This wide range of coexistence rates has been largely attributed to differences in methods, definitions, and diagnostic criteria [7]. Additionally, only a few studies report the incidence of cases of DDH that require treatment in patients with CMT. If only such cases were considered, the incidence rates range from 0 to 8.5 % [3, 6, 7, 13, 19].

The indications for hip imaging in the setting of CMT remain controversial. Hummer and MacEwen [12] were the first to recommend clinical and roentgenographic examination of both hips in all children with CMT, which was again recommended in a later study by Morrison and MacEwen [15]. Tien et al. [7] conducted an ultrasonographic study of the coexistence of CMT and DDH and recommended that ultrasound imaging of the hip be performed routinely for patients with CMT. von Heideken et al. [8] also recommend that children with CMT be evaluated for DDH but did not differentiate between physical exam and radiographic screening. Most recently, Kim et al. [6] and Minihane et al. [19] came to the conclusion that bilateral hip ultrasound should not be recommended routinely for patients with CMT, given that, in their series, all patients with DDH requiring treatment also had an abnormal clinical hip examination.

There is no consensus on when imaging of the hips is indicated in patients with CMT. The American Academy of Pediatrics [20] recommends the screening of newborn infants using physical examination. While the American Academy of Pediatrics recommends considering imaging in addition to physical exam for patients with a breech presentation or positive family history, it is not recommended for routine screening in cases of CMT. The Pediatric Orthopaedic Society of North America agreed with these recommendations in their statement regarding current guidelines for DDH screening. The United States Preventative Services Task Force [22] and the Cochrane Collaboration [23] conducted systematic reviews on the literature regarding screening for DDH in infants and found that the existing evidence ranges from fair to poor and is insufficient to give clear and conclusive recommendations for practice. Additional guidelines exist from the American College of Radiology [24, 25], but none of these recommendations address routine screening in patients with CMT.

The purpose of this study is to determine (1) the coexistence rate of DDH requiring treatment in patients with the diagnosis of CMT who have had hip imaging at our institution and (2) whether there are patients with CMT and DDH requiring treatment who present with a normal physical exam of the hip.

## Methods

After approval for the study was obtained from our institutional review board, we identified patients diagnosed with CMT between 1/1/2003 and 9/1/2012 at our institution. Patients were excluded if they did not have hip imaging, or if the torticollis was associated with a neuromuscular or syndromic etiology, congenital anomaly, or ocular problem. Charts were reviewed for the reason for referral, demographic information, family history, physical exam findings, imaging results, treatment for DDH (where applicable), and complications. Descriptive statistics were calculated for analysis.

## Results

**Fig. 1:**
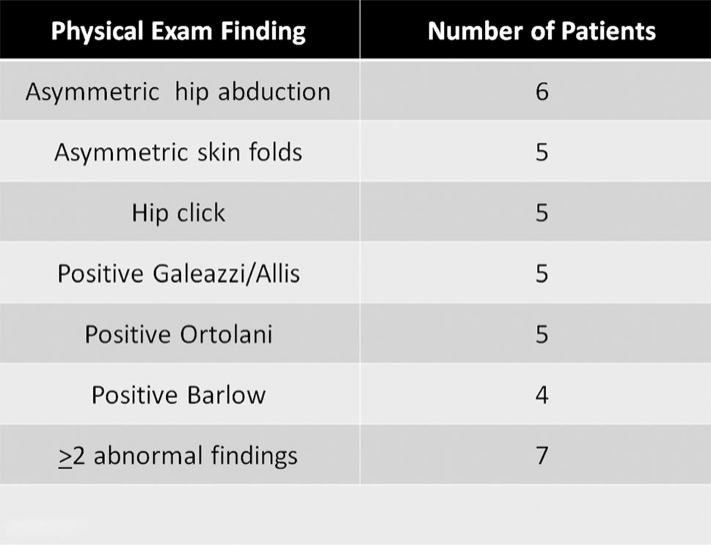
Abnormal findings on physical exam of the hips in patients with congenital muscular torticollis (CMT). Used with the permission of the Children’s Orthopaedic Center, Los Angeles

Ninety-seven patients met the inclusion criteria, 55 % (53/97) females and 45 % (44/97) males, with an average age at presentation of 7.9 months (range 0.3–27.1 months). On physical exam of the hips, 20 % (19/97) of patients had an abnormality detected, including asymmetric abduction, asymmetric skin folds, hip click, positive Galeazzi/Allis, positive Ortolani, and positive Barlow (see Fig. 1). Seven patients had two or more of the above positive findings on physical exam. Ultrasound was the first hip imaging performed in 28/97 (29 %) patients at an average age of 2.5 months (range 0.4–5.9 months). Plain radiographs were the first hip images performed in 69/97 (71 %) patients at an average age of 11.2 months (range 2.0–40.2 months). Despite the abnormal hip exam, 10 of these 19 patients did not have any evidence of hip dysplasia on imaging.

**Fig. 2:**
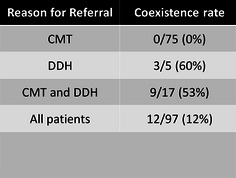
Patients with CMT who were found to have DDH, separated by reason for referral. Used with the permission of the Children’s Orthopaedic Center, Los Angeles

**Fig. 3:**
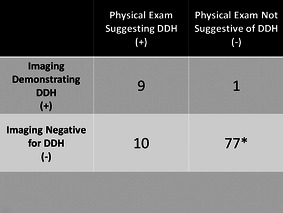
Table showing the rate of true-positive, false-positive, true-negative, and false-negative physical exam findings using the data from our series. Of note, this table excludes patients who did not have hip imaging performed. *Includes two patients who were previously treated for developmental dysplasia of the hip (DDH) and, at the time of presentation, had no clinical or radiographic findings suggestive of DDH. Used with the permission of the Children’s Orthopaedic Center, Los Angeles

**Fig. 4:**
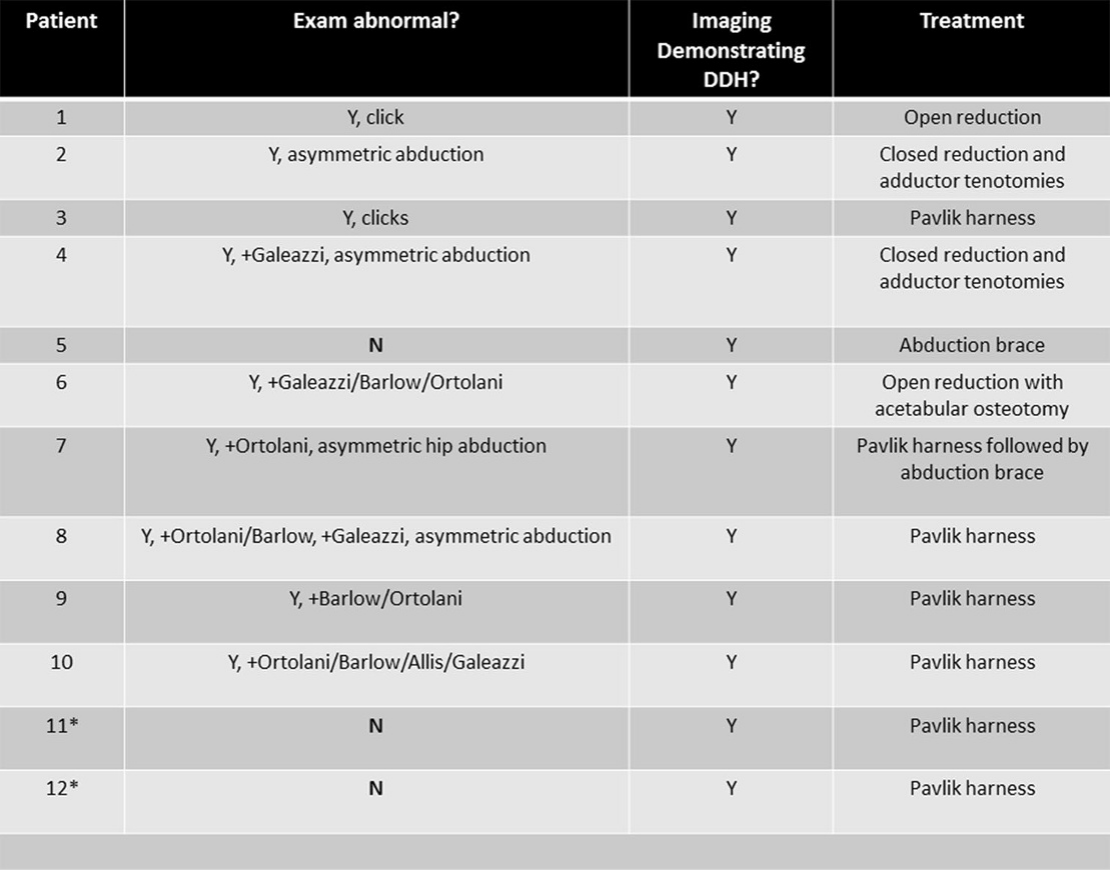
Patients with DDH demonstrating (1) physical exam abnormalities, (2) radiographic abnormalities, and (3) treatment received. *Presented as second opinion, previously treated. Used with the permission of the Children’s Orthopaedic Center, Los Angeles

Of the 97 patients diagnosed with CMT that had hip imaging available, 12 % (12/97) were found to have hip dysplasia that required treatment. The rate of patients with CMT found to have DDH listed by reason for referral may be seen in Fig. 2. Of the patients who were diagnosed with DDH, 75 % (9/12) had an abnormal physical exam. Of the three patients with a normal exam, two patients were being seen as a second opinion and had a past medical history of DDH that had already been successfully treated with a Pavlik harness. Only one patient who had a normal physical exam was diagnosed with DDH at that time. Therefore, of the patients who had DDH at the time of presentation, 90 % (9/10) had an abnormal hip exam (see Fig. 3). All 12 of the patients with the diagnosis of DDH received treatment, including Pavlik harness only (6), abduction brace only (1), Pavlik harness followed by abduction brace (1), closed reduction and adductor tenotomies (2), and open reduction (2) with acetabular osteotomy (1) (see Fig. 4).

## Discussion

Of the 97 patients diagnosed with CMT that had hip imaging available, 12 % (12/97) were found to have hip dysplasia that required treatment. This falls within the wide range of rates previously reported in the literature, 0–20 % [1–19], but is higher than rates where only cases of DDH that required treatment were included, 0–8.5 % [3, 6, 7, 13, 19]. The wide variability in coexistence rates had been largely attributed to differences in methods, definitions, and diagnostic criteria [7], and this study is no exception. Additionally, this rate may be higher because we did not include patients with CMT who did not have hip imaging available in our system, which may have lowered the rate. However, one of our goals of the study was to determine whether there are patients with CMT who require hip imaging to reveal DDH requiring treatment (versus clinical exam alone); therefore, we decided to exclude those patients without imaging. Despite the wide range of coexistence rates reported in the literature, there is a growing body of evidence supporting the existence of an association between CMT and DDH, which is important for the clinician to be aware of when treating patients with either of these diagnoses.

There is general agreement that patients with CMT should be screened for DDH [6–8, 12, 15, 19]. However, there is no consensus as to whether routine screening should consist of physical exam and imaging of the hips, or physical exam alone. The most recent studies suggest that physical exam alone is sufficient screening, given that all the patients in their series with DDH had an abnormal clinical hip exam [6, 19]. Of the 12 % of patients with CMT and DDH, only 75 % (9/12) of our patients with DDH had an abnormal clinical hip exam. However, excluding the two patients who were previously treated for DDH, nine out of ten patients (90 %) with DDH had an abnormal physical exam. The argument could be made that adding imaging of the hips to routine screening is unnecessary, since the overwhelming majority present with abnormal physical exam findings. However, there was one patient in our series who had DDH that required treatment who did not have any abnormalities on clinical exam of the hip. Although patients like this may be a rarity, stating that screening hip imaging in patients with CMT is not necessary may lead to a small number of cases of DDH that go undiagnosed.

Due to the fact that there were many patients in this series who were excluded due to the lack of hip imaging, we cannot establish a true false-negative rate. Nevertheless, the presence of any false-negative exam supports the need for radiographic screening in this high-risk patient population.

## Conclusion

There are a number of recommendations in the literature for the screening of developmental dysplasia of the hip (DDH), including those from the American Academy of Pediatrics, the United States Preventative Services Task Force, the American College of Radiology, and the Pediatric Orthopaedic Society of North America. Recommendations in these guidelines include risk factors such as female gender, breech presentation, and a positive family history, plus physical exam findings. None of these guidelines address screening for DDH specifically in patients with congenital muscular torticollis (CMT). There is a clear association demonstrated in this and other series between DDH and CMT. Given the fact that it is possible for even an experienced examiner to miss DDH on physical exam alone, we recommend either an ultrasound in patients less than 6 months of age or an anteroposterior pelvis radiograph in those over 6 months of age in this high-risk population.
